# Halogen-Induced Controllable Cyclizations as Diverse Heterocycle Synthetic Strategy

**DOI:** 10.3390/molecules25246007

**Published:** 2020-12-18

**Authors:** Hideyasu China, Ravi Kumar, Kotaro Kikushima, Toshifumi Dohi

**Affiliations:** 1 Department of Medical Bioscience, Nagahama Institute of Bio-Science and Technology, 1266, Tamuracho Nagahama-shi, Shiga 526-0829, Japan; 2 Department of Chemistry, J. C. Bose University of Science & Technology, YMCA, NH-2, Sector-6, Mathura Road, Faridabad, Haryana 121006, India; ravi.dhamija@jcboseust.ac.in; 3 College of Pharmaceutical Sciences, Ritsumeikan University, 1-1-1 Nojihigashi, Kusatsu, Shiga 525-0058, Japan; kixy@fc.ritsumei.ac.jp

**Keywords:** intramolecular cyclization, halocyclization, halogen intermediate, reagent switch, organocatalyst, substrate switch, *endo*/*exo* selectivity

## Abstract

In organic synthesis, due to their high electrophilicity and leaving group properties, halogens play pivotal roles in the activation and structural derivations of organic compounds. Recently, cyclizations induced by halogen groups that allow the production of diverse targets and the structural reorganization of organic molecules have attracted significant attention from synthetic chemists. Electrophilic halogen atoms activate unsaturated and saturated hydrocarbon moieties by generating halonium intermediates, followed by the attack of carbon-containing, nitrogen-containing, oxygen-containing, and sulfur-containing nucleophiles to give highly functionalized carbocycles and heterocycles. New transformations of halogenated organic molecules that can control the formation and stereoselectivity of the products, according to the difference in the size and number of halogen atoms, have recently been discovered. These unique cyclizations may possibly be used as efficient synthetic strategies with future advances. In this review, innovative reactions controlled by halogen groups are discussed as a new concept in the field of organic synthesis.

## 1. Introduction

Halogen sources, such as chlorine, bromine, and iodine, are present in high abundance on the earth and are indispensable in organic synthesis since they enhance the reactivity of organic compounds. The use of halogen-containing reagents and oxidants in modern chemistry means that the environmental impact of a reaction is lowered when compared with the use of heavy metals, thus, expanding the field of activity for the synthesis of cyclic compounds. The intramolecular cyclization that accompanies the introduction of halogens is attractive from the viewpoint of green and process chemistry perspectives. In the formation of lactones and lactams, halogen-induced intramolecular cyclizations are usually carried out under mild conditions, while condensation strategies for intramolecular cyclization mostly require the use of water-sensitive reagents such as Lewis acids and condensation reagents, under heating conditions. In addition, the use of halogen-induced cyclizations avoids the occurrence of undesirable intermolecular bimolecular side-reactions. The halogen-containing molecules that result from intramolecular halocyclizations are also advantageous as they can be further functionalized.

Halogen-induced intramolecular cyclizations have a long history of being used for the transformation of a linear to a cyclic molecule. In the late 19th century, the first halogen-induced intramolecular cyclization was reported in the form of the bromolactonization of olefinic acids [1,2]. In the middle of the 20th century, *endo*/*exo*-cyclized isomers were identified [3]. In the latter half of the 20th century, the halolactonizations of olefinic acids and amides, and the haloetherifications of olefinic alcohols were reported [4,5,6,7]. However, in the 21st century, there have been significant developments in halogen chemistry, as halogens have been determined to contribute to the control of high selectivities for the cyclization reactions [8,9,10,11,12,13,14,15,16,17,18,19,20]. In modern halogen chemistry, halogen-controlled reaction strategies that can contribute toward selective transformations have, thus, become an important issue and are of utmost interest in organic synthesis.

**Figure 1:**
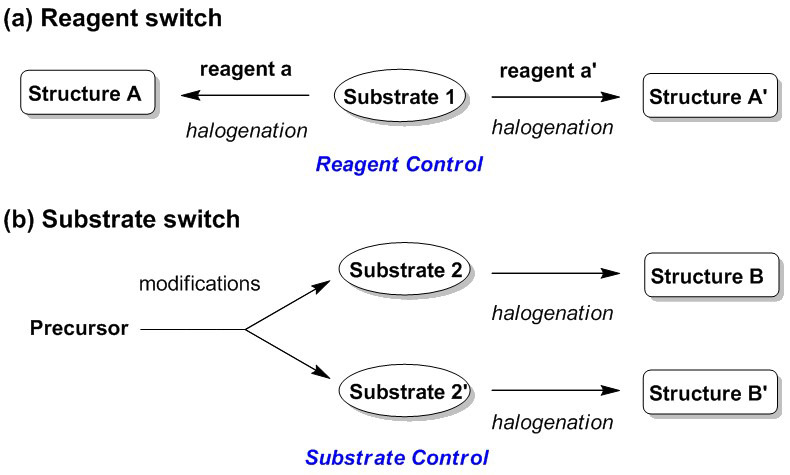
Overall concepts of controllable syntheses: categorized into (**a**) reagent-switchable and (**b**) substrate-switchable strategies.

Halolactonizations of olefinic carboxylic acids are known as being typically controllable intramolecular cyclization reactions that enable diverse heterocycle formations. In this review, these reactions are categorized into (a) reagent-switchable reactions and (b) substrate-switchable reactions (Figure 1). Reagent-switchable cyclization reactions allow the selective synthesis of two or more products from one substrate via reagent control (Figure 1a), whereas substrate-switchable reactions can be defined as a synthetic sequence in which the final products can be altered, according to the pre-modifications and/or isomerisms of the substrates, e.g., protection of amines and the *cis*-*trans* isomerization of olefins (Figure 1b). These controllable intramolecular cyclizations are excellent for the selective synthesis of isomers and their derivatives.

**Figure 2:**
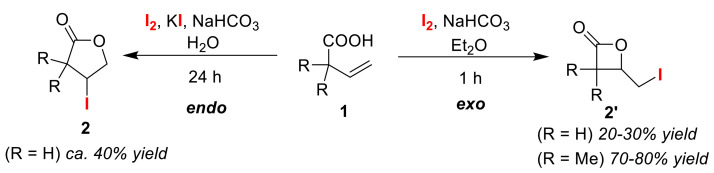
An early reported reagent-switchable reaction enabling *endo*/*exo* selective iodolactonization.

An early example of a reagent-switchable reaction is the cyclization involved in iodolactonization reactions. In 1953, Tamelen and Shamma reported the iodolactonization of olefinic acid **1** to obtain γ-lactone **2** (Figure 2) [3]. In 1972, Barnett and Sohn then reported the selective iodolactonization of the same olefinic acid **1** to alternatively synthesize β-lactone **2′** [21]. From these studies, the *endo*/*exo* selectivity of iodolactonizations was found to be strongly influenced by the reaction conditions and combination of the reagents.

**Figure 3:**
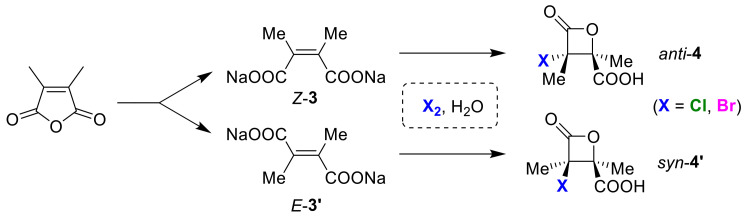
An early example of substrate-switchable cyclization for stereo-selective halo-lactonizations.

However, an early example of a substrate-switchable cyclization was observed in chloro-lactonization and bromo-lactonization reactions. In 1937, the first example of selective β-lactone formation leading to *anti*-**4** and *syn*-**4′** from *Z*-**3** and *E*-dicarboxylates **3′**, respectively by means of a halolactonization reaction was reported by Tarbell and Bartlett [22]. In 2001, the William’s group re-investigated this study to support the regiospecific halo-lactonizations of *Z*-**3** and *E*-dicarboxylate **3′** to *anti-***4** and *syn*-lactone **4′**, respectively [23] (Figure 3). It should be noted that the *syn*/*anti* selectivity in halo-lactonizations clearly depends on the isomerisms of substrates that can be prepared from common synthetic precursors.

**Figure 4:**
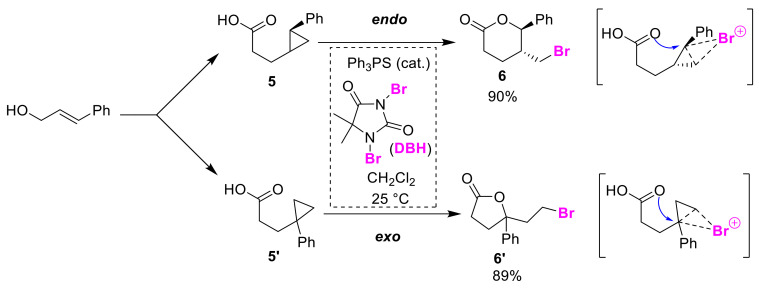
Regioisomer-dependent *endo*/*exo*-selective bromolactonizations of cyclopropyl carboxylic acids **5** and **5′**.

Halogen-addition type intramolecular cyclizations of unsaturated substrates, such as the halo-lactonization of olefinic acids, are representative examples of controllable syntheses, while those for saturated substrates, such as the halo-lactonization of cyclopropyl carboxylic acids **5** and **5′** using a Lewis base sulfide catalyst and 1,3-dibromo-5,5-dimethylhydantoin (DBH), are in the minority, but are still noteworthy (Figure 4) [24]. The Yeung group revealed that the bromo-lactonization of *trans*-1,2-disubstituted cyclopropyl carboxylic acid **5** and 1,1-disubstituted isomer **5′** selectively yields *endo*-cyclized **6** and *exo*-isomer **6′**, respectively, according to the substitution patterns of substrates **5** and **5′**. In this reaction, the cyclopropane moiety acts as an electron acceptor for the electrophilic brominations.

**Figure 5:**
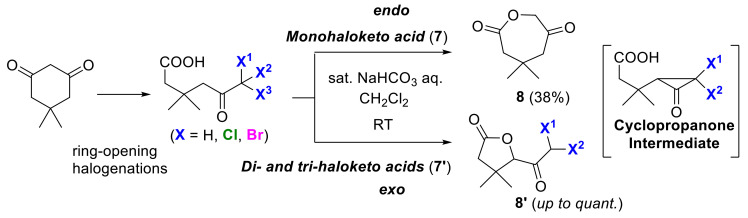
*Endo*/*exo*-selective dehalolactonization reactions of haloketo acids **7** and **7′** controlled by the internal halogen groups.

Meanwhile, halogen-elimination type intramolecular cyclizations are rather rare, especially in the form of controllable strategies. We recently discovered the unique halogen-controlled dehalolactonization reaction of haloketo acids **7** and **7′** during our investigation on the monochlorodimedone assay targeting haloperoxidase [25]. Dehalolactonization reactions of these haloketo acids proceed via a unique cyclopropanone intermediate (Figure 5), which then allows the *endo*-cyclization or *exo*-cyclization products to be produced selectively. Although *endo*-cyclization of monohaloketo acids **7** gave oxolactones **8**, *exo*-cyclization of di-haloketo and tri-haloketo acids **7′** instead produced haloacyl lactones **8′**. Using this transformation, the ring structure of the final products differed according to the number of pre-installed halogen groups.

Controllable cyclizations of linear molecules are, thus, useful skeleton formations that allow great versatility in synthetic chemistry. Highly stereoselective reactions using organocatalysts have recently attracted significant attention in the literature [1,26,27,28,29,30,31,32,33,34,35,36,37,38,39,40]. However, the focus of this review is on reactions that involve selective control of the skeleton formations of the molecule such as control over endo/exo-cyclized products. Halogen-induced controllable cyclizations can be categorized into reagent-switchable and substrate-switchable approaches, and these can be further classified into (1) endo/exo selective cyclizations, which are important for control of the size of rings, (2) O/N atom-selective cyclizations to introduce heteroatoms, (3) ene/diene selective cyclizations related to aromatization, (4) syn/anti (cis/trans) selective cyclizations for constructing diastereomers, (5) enantioselective cyclizations leading to enantiomers, and (6) other miscellaneous reactions.

## 2. Reagent-Switchable Cyclizations

Intramolecular cyclization is one of the important keys in constructing the core skeleton of organic molecules. Switchable intramolecular cyclizations allow the selective synthesis of two or more products from one substrate via control of the reagents. In the initial stage of the reaction, a halogenating agent functions as a reaction initiator to generate a reactive intermediate that has selectivity potential. Although catalysts may sometimes play pivotal roles in terms of high selectivity, it is worth mentioning that reaction conditions, such as solvent and base, also strongly influence product selectivity.

### 2.1. Endo/Exo Selective Cyclizations

In an iodolactonization reaction of olefinic acid **1** as a classical switchable reaction, γ-lactone **2**, and β-lactone **2′** can be selectively synthesized (Figure 2). Exo-cyclization that gives β-lactone **2′** proceeds via kinetic control on a short time scale, while endo-cyclization that produces γ-lactone **2** proceeds via thermodynamic control on a long-time scale. This is due to the slow interconversion of β-lactone **2′** to γ-lactone **2**. However, styryl acetate only promotes endo-cyclization despite it taking place on a short time scale. This can be interpreted as being the result of the stabilization of a carbocation intermediate due to the effect of the γ-phenyl substituent. The study showed that the time-controlled strategy in endo/exo selectivity is strongly constrained by the reactivity of the substrates.

**Figure 6:**
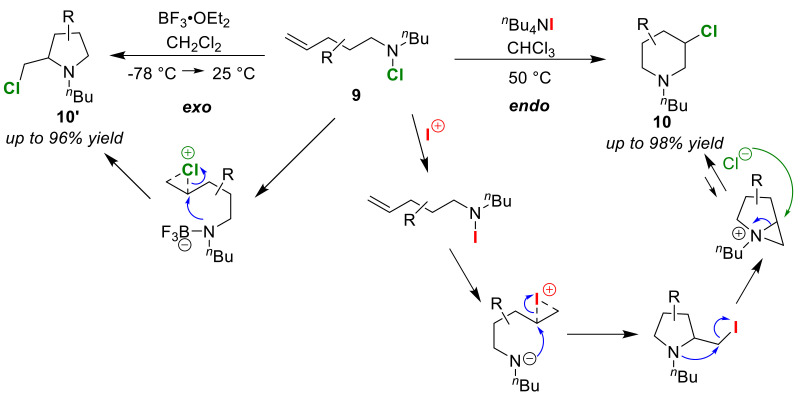
*Endo*/*exo* selective chloro-amino-cyclization reactions of olefinic *N*-chloro-amines **9**.

Göttlich and co-workers achieved the selective synthesis of 3-chloro-piperidines **10** and 2-chloromethyl-pyrrolidines **10′** from the chloroaminocyclization of olefinic *N*-chloroamines **9** (Figure 6) [41,42]. Endo-cyclization with *^n^*Bu_4_NI gave the piperidines **10**, whereas exo-cyclization with BF_3_·OEt_2_ instead gave the pyrrolidines **10′**. In terms of endo-cyclization, the authors proposed a reaction mechanism that proceeds via an aziridinium ion intermediate followed by an iodo-amino-cyclization reaction. The intermediate and product **10** are present in equilibrium, and both substrate **9** and product **10** serve as bases. It has already been reported that pyrrolidine **10′**, which is an exo-cyclized product, is converted to piperidine **10** via a rearrangement under basic conditions [43,44]. Unfortunately, the generation of an *N*-iodoamine intermediate cannot be detected because the reactivity of *N*-iodoamine is too high.

**Figure 7:**
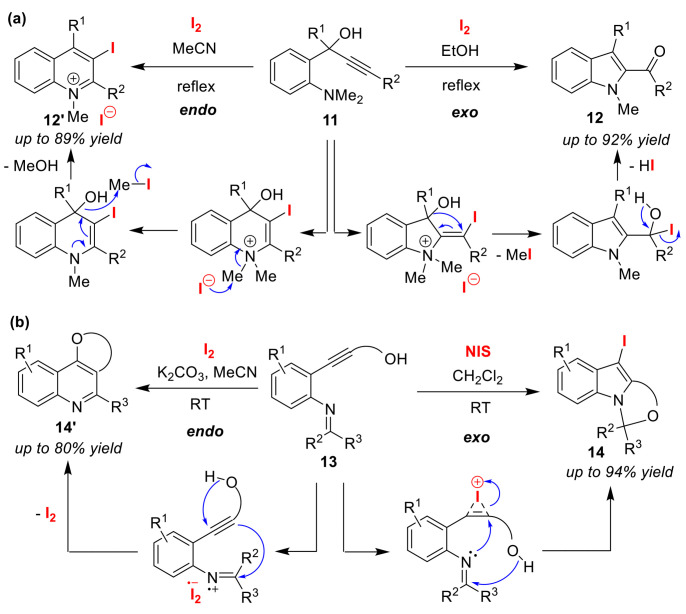
Endo/exo selective iodo-amino-cyclizations of (**a**) *N*,*N*-dimethylaniline with a propargyl alcohol moiety **11** and (**b**) alkynylphenylimines bearing nucleophilic hydroxy groups **13**.

Flynn and co-workers achieved the selective synthesis of indoles **12** and quinolines **12′** from *N*,*N*-dimethylanilines **11** that feature a propargyl alcohol moiety (Figure 7a). The *exo*-cyclization reaction to produce indoles **12** proceeds in a protic solvent such as EtOH or MeOH, while endo-cyclization occurs in an aprotic solvent such as MeCN or CH_2_Cl_2_ to alternatively give quinolines **12′** [45]. Although the key factor in the selectivity is the solvent, its control mechanism has not been clarified. Further, the authors extended this method to the synthesis of tricyclic compounds [46,47]. This synthesis is accomplished using two types of iodine electrophiles to trigger a domino cascade reaction of alkynylphenylimines **13** bearing nucleophilic hydroxy groups. However, aprotic solvents are used in the formation of the skeleton of both indoles **14** and quinolines **14′**, and the control factors are, thus, different from those described for the previous method including the use of the iodination agents, I_2_ and *N*-iodosuccinimide (NIS). The authors proposed that I_2_ can interact with the imine site, whereas NIS activates the alkyne site (Figure 7b). It was explained that the cationic carbon atom of the imine initiates quinoline formation via electrophilic cyclization involving the alkyne, while the activated alkyne site forms an indole skeleton via a nucleophilic cyclization with the imine nitrogen atom.

**Figure 8:**
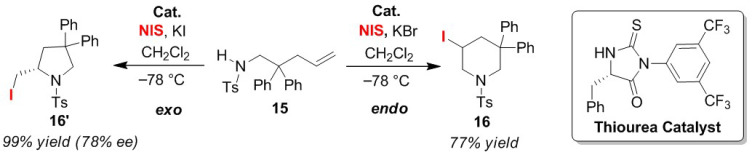
Endo/exo selective iodo-amino-cyclization reactions of olefinic amines **15** using a thiourea catalyst.

The Wirth group reported an effective organocatalytic approach for the selective endo/exo-cyclization of olefinic *N*-tosylamines **15** [48]. In the presence of chiral cyclic thiourea as an organocatalyst, a catalytic amount of KBr was added to give 3-iodopiperidine **16**, the endo-cyclization product of olefinic *N*-tosylamine (Figure 8). However, the addition of a catalytic amount of KI afforded 2-iodomethyl-pyrrolidine **16′** stereo-selectively as the exo-cyclized product. From this study, it was, therefore, confirmed that these additives affect the halogen bonding interactions of the thiourea catalyst toward substrates and electrophilic iodine species. However, the precise control mechanism of the endo/exo selectivity still remains somewhat unclear.

### 2.2. O/N Atom-Selective Cyclizations

**Figure 9:**
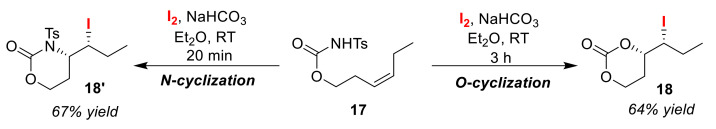
Time-dependent *O*/*N* atom-selective iodo-cyclizations of olefinic *N*-tosyl carbamates **17**.

One of the most attractive extensions of switchable halocyclizations is the incorporation of heteroatoms into the ring structure. Hence, C–N bond formation that occurs during the cyclization of olefinic carbamates give rise to urethane derivatives, while C–O bond formation produces carbonate derivatives. Hirama and co-workers found that *N*-cyclization and *O*-cyclization modes in the iodo-cyclizations of olefinic *N*-tosyl carboxamides **17** can be successfully controlled by adjusting the reaction time (Figure 9) [49]. In a two-phase system consisting of Et_2_O and aqueous NaHCO_3_, the treatment of olefinic *N*-tosyl carbamate **17** with I_2_ for 20 min generated only cyclic urea **18′**, while cyclic carbonate **18** was exclusively obtained after a prolonged reaction time of 3 h.

**Figure 10:**
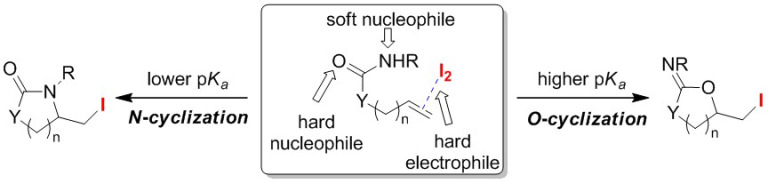
HSAB control in the O/N atom-selective cyclization of amphoteric nucleophiles.

**Figure 11:**
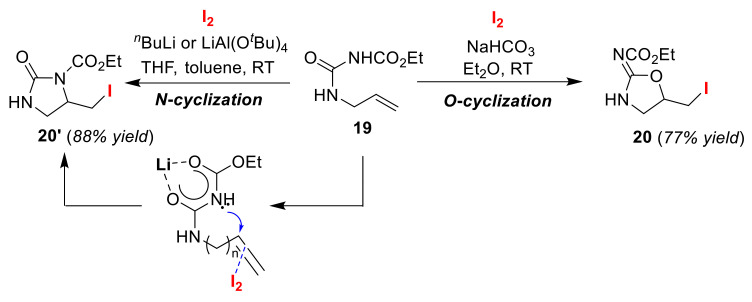
O/N atom-selective iodo-cyclizations of olefinic carbamates **19**.

Typical iodo-cyclizations with amphoteric nucleophiles, such as carbamates, amides, or ureas, preferentially gave the corresponding *O*-cyclization products over the *N*-cyclized counterparts. The reason for this product control can be explained using the hard and soft (Lewis) acids and bases (HSAB) principle (Figure 10). The electronegative O atom, rather than the N atom, shows a preference for attacking iodine-olefin π complexes, which are characterized as hard electrophiles. However, in order to promote the *N*-cyclization reaction, it is necessary to lower the p*Ka* value at the NH position via *N*-substitution. The Taguchi group succeeded in the *O*/*N* atom-selective cyclization of olefinic urea **19** using a carbamate protection group [50]. Although the selective *O*-cyclization reaction to obtain *N*-2-oxazolidinylidene **20** proceeds under moderate conditions, selective *N*-cyclization to afford 2-imidazolidinone **20′** was achieved using a metal base, such as *^n^*BuLi or LiAl(O*^t^*Bu)_4_ (Figure 11). Similar bases are used for the selective *N*-cyclization of olefinic carbamates and amides [51]. It is considered that the *N*-cyclization preferentially proceeds because the nucleophilicity of the O atom is decreased due to the formation of a six-membered chelate ring structure.

**Figure 12:**
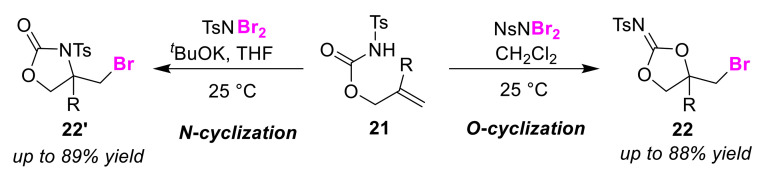
O/N atom-selective bromocyclizations of olefinic *N*-tosylcarbamates **21**.

Zhou and co-workers developed an O/N regioselective bromocyclization method to produce olefinic *N*-tosyl carbamates **21** [52]. The outcome of this reaction is heavily dependent on the electrophilic source used. NsNBr_2_ selectively produces the *O*-cyclization products **22** in CH_2_Cl_2_ (Figure 12). However, selective *N*-cyclization products **22′** can be obtained by treating TsNBr_2_ with *^t^*BuOK in tetrahydrofuran (THF), but the control mechanism of this reaction remains unclear.

**Figure 13:**
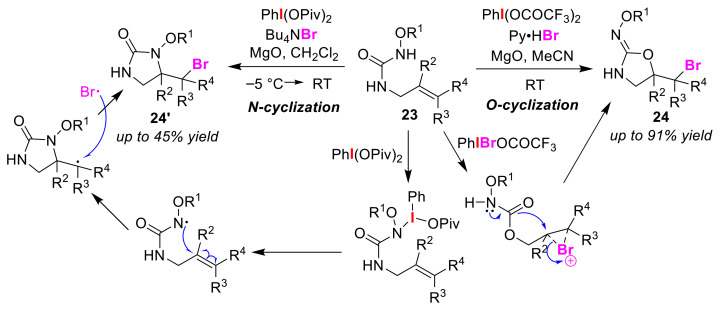
O/N atom-selective bromocyclizations of olefinic *N*-substituted ureas **23**.

The Cariou group achieved the O/N atom-selective bromocyclizations of olefinic ureas **23** using hypervalent iodine reagents [53]. *O*-cyclization with PhI(OCOCF_3_)_2_ and the bromine source Py·HBr gave oxazolidinone oximes **24**, while *N*-cyclization with PhI(OPiv)_2_ and *^t^*Bu_4_NBr produced *N*-hydroxylated 2-imidazolidinone **24′** (Figure 13). In the *O*-cyclization, an ionic mechanism in which PhI(Br)OCOCF_3_ as a bromonium cation is generated by the ligand exchange of PhI(OCOCF_3_)_2_ has been proposed. In the *N*-cyclization, a radical mechanism that proceeds via the formation of an N–I bond intermediate from PhI(OPiv)_2_ and an oxime moiety is believed to occur. A synthetic method for synthesizing cyclic ureas and cyclic iso-ureas has also been reported to proceed via an O/N atom-selective cyclization using a combination of PhI(OAc)_2_ and a metal catalyst [54].

**Figure 14:**
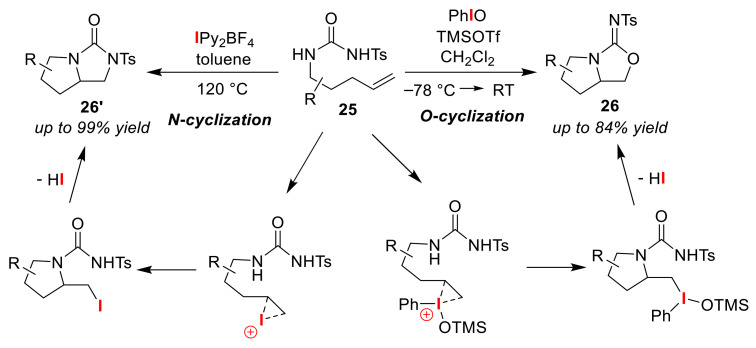
Synthesis of bicyclic iso-ureas **26** and ureas **26′** via *O*/*N* atom-selective iodo-cyclizations of olefinic *N*-tosyl-ureas **25** using different active iodine species.

In addition, in terms of *O*-cyclization, Cochran and Michael used a hypervalent iodine reagent, iodosobenzene (PhIO), in an intramolecular oxo-amination reaction to achieve the selective synthesis of bicyclic iso-ureas **26** from olefinic *N*-tosylureas **25** [55]. The authors proposed a reaction mechanism in which the iodine(III) reagent, PhIOTMS(OTf), derived from the reaction of PhIO with trimethylsilyl triflate (TMSOTf) contributes to the generation of an iodonium(III) ion (Figure 14). In this reaction mechanism, it can be determined that the selective *O*-cyclization reaction proceeds via a second intermediate generated by the 5-exo-cyclization of the nitrogen at the urea moiety of the iodonium intermediate. Due to the high nucleophilicity of the oxygen atom, *O*-cyclization preferentially takes place under strongly acidic conditions. *O*-cyclization is favored even more under weakly acidic conditions, such as in acetic acid, but the product yield is very low. On the other hand, Muñiz and co-workers selectively synthesized *N*-cyclized bicyclic ureas **26′** via an intramolecular diamination reaction using IPy_2_BF_4_ [56]. Their method, in which a highly reactive iodonium intermediate is proposed, achieves excellent yield and high selectivity, despite high temperature conditions being used. A method for synthesizing a similar bicyclic compound via temperature-dependent O/N atom-selective cyclization has also been reported [57].

**Figure 15:**
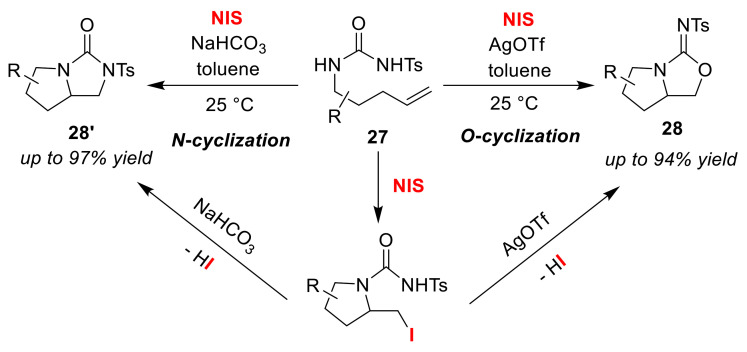
Synthesis of bicyclic iso-ureas **28** and ureas **28′** via the O/N atom-selective iodo-cyclizations of olefinic *N*-tosyl-ureas **27** using NIS and additives.

Li and Widenhoefer developed the O/N atom-selective cyclizations of olefinic *N*-tosyl-ureas **27** using NIS [58]. Reaction conditions using AgOTf gave bicyclic isoureas **28** via *O*-cyclization, whereas the use of NaHCO_3_ instead afforded bicyclic ureas **28′** via *N*-cyclization (Figure 15). In either case, the iodo-amino-cyclization reaction in the first step produces a monocyclic intermediate, and the additive strongly affects the O/N selectivity at a second cyclization step. It is worth mentioning that AgOTf efficiently promotes a second-stage nucleophilic substitution-type cyclization. The corresponding bromo-substituted version has been reported, which shows the same trend in selectivity [59].

### 2.3. Ene/Diene Selective Cyclizations

**Figure 16:**
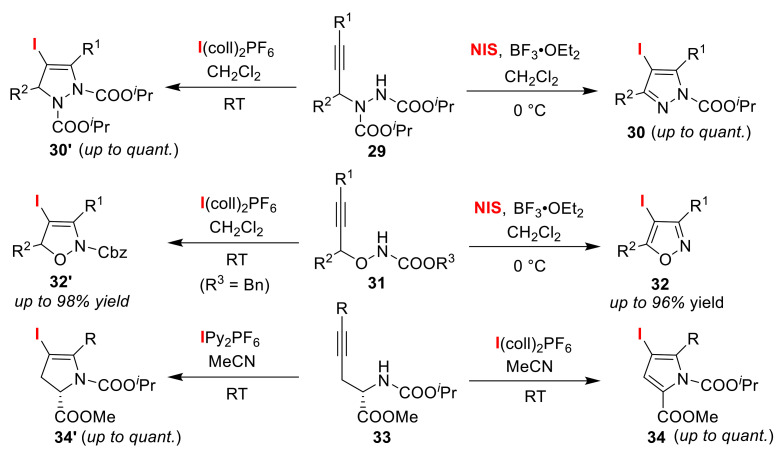
Selective syntheses of five-membered heterocycles via the iodo-cyclization of propargylic hydrazides **29**,*N*-alkoxycarbonyl propargylic hydroxylamines **31**,and α-propargylic glycines **33**.

**Figure 17:**
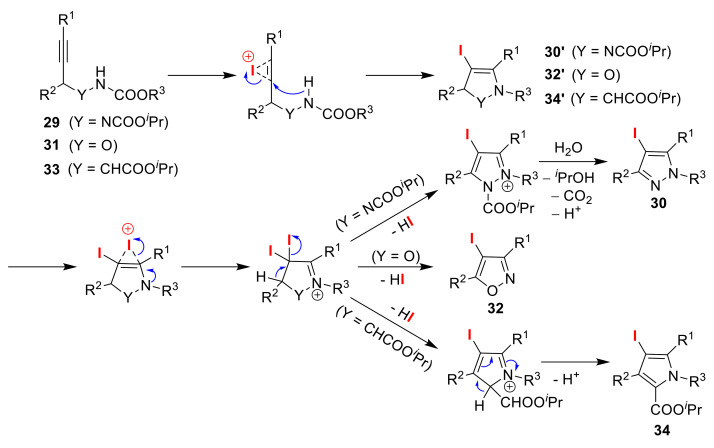
Plausible reaction mechanism of the iodo-cyclizations of alkynyl carbamates (**29**, **31**, and **33**).

The Wada group synthesized different five-membered heterocycles via the iodination of alkynyl carbamates (Figure 16). Applying NIS to propargylic hydrazides **29** in the presence of BF_3_·OEt_2_ afforded pyrazoles **30**, while the use of bis(2,4,6-collidine)iodonium(I) hexafluorophosphate (I(coll)_2_PF_6_) gave dihydropyrazoles **30′** [60]. Similar treatment of *N*-alkoxycarbonyl propargylic hydroxylamines **31** selectively yielded isoxazoles **32** and 2,5-dihydroisoxazoles **32′** [61]. Although the application of I(coll)_2_PF_6_ to α-propargylic glycine **33** gave pyrroles **34**, the use of bis(pyridine)iodonium(I) hexafluorophosphate (IPy_2_PF_6_) resulted in 2,3-dihydropyrroles **34′** [62]. In the course of three switchable reactions, the authors proposed that the 5-endo-cyclization of iodonium ion intermediates, generated by the electrophilic addition of iodine cations to alkyne moieties, gave heterocycles **30′**, **32′**, and **34′**. These heterocycles then underwent deiodination, which was followed by iodination to produce the aromatized products **30**, **32**, and **34**, respectively (Figure 17).

**Figure 18:**
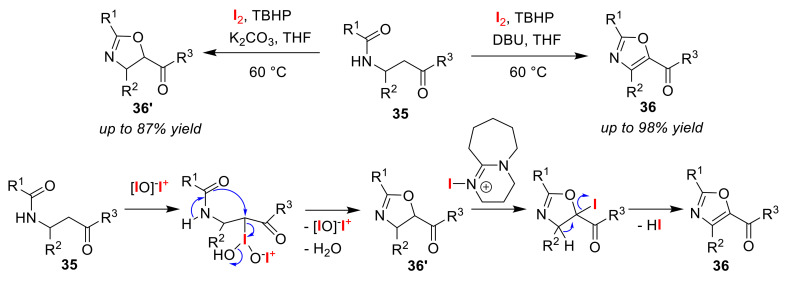
Selective synthesis of oxazoles **36** and oxazolines **36′** via the oxidative cyclization of β-acylamino ketones **35** using I_2_ and TBHP under basic conditions.

Gao and co-workers achieved the base-dependent selective synthesis of oxazoles **36** and oxazolines **36′** via the oxidative cyclization of β-acylamino ketones **35** in the presence of an iodine catalyst [63]. When K_2_CO_3_ was used as a base, oxazolines **36′**, in the form of enes, were produced. In contrast, treatment with 1,8-diazabicyclo[5.4.0]undec-7-ene (DBU) instead of K_2_CO_3_ led to the formation of oxazoles **36**, in the form of aromatics. The authors proposed a reaction mechanism in which the active high-valent iodine species, [IO]^−^I^+^ generated from *tert*-butyl hydroperoxide (TBHP), and I_2_ under basic conditions, was introduced to the substrates, triggering intramolecular nucleophilic substitution (Figure 18). The *O*-cyclization reaction at the amide moiety, thus, gave oxazoline products **36′**. In addition, the authors explained that DBU promotes further iodination and subsequent deiodination for the production of oxazoles **36**.

**Figure 19:**
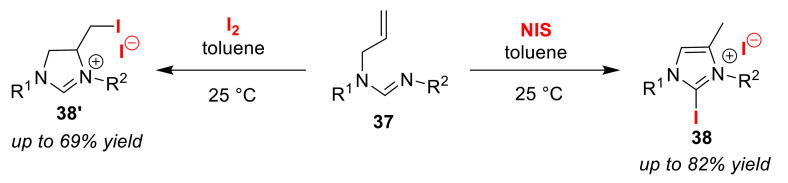
The selective synthesis of cyclic formamidinium salts **38** and **38′** via iodo-amino-cyclization of *N*-alkenyl formamidines **37** using iodine reagents.

The Zhang group developed the selective synthesis of two cyclic formamidinium salts via the iodo-amino-cyclization of *N*-alkenyl formamidines **37** in the presence of an iodinating agent [64]. Imidazoles **38** were obtained upon treatment with NIS, while the formation of non-aromatic heterocycles **38′** instead proceeded via iodo-amino-cyclization using iodine (Figure 19). They also demonstrated that 6-membered and 7-membered rings could be formed using the same method.

### 2.4. Syn/Anti Selective Cyclizations

**Figure 20:**
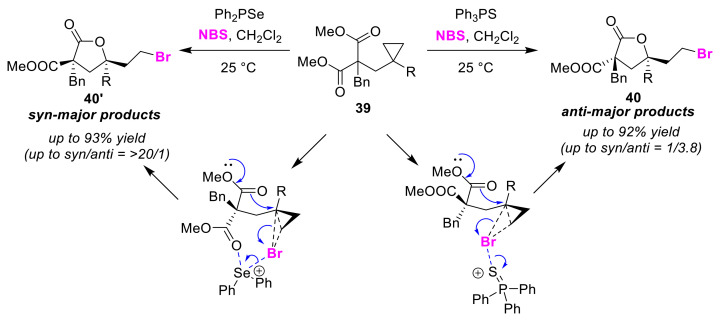
*Syn/anti* selective bromolactonizations of cyclopropyl diesters **39** using chalcogenide catalysts.

Yeung and co-workers reported switchable *syn*/*anti* selective bromolactonizations of cyclopropyl diesters **39** [65]. The use of cyclopropanes as substrates in bromocyclizations is noteworthy in this study in which the chalcogenide catalysts, Ph_3_PS and Ph_2_PSe, play important roles in the activation of the cyclopropane moiety in the substrates. In this method, the use of Ph_3_PS in the presence of *N*-bromosuccinimide (NBS) gave anti-form lactones **40**, whereas the use of Ph_2_PSe gave syn-form lactones **40′** (Figure 20). Although a detailed reaction mechanism remains to be revealed, it was suggested that the observed anti-selectivity might be influenced by the interaction that takes place between the Ph_2_Se chalcogenide catalyst and the ester group in the substrate.

### 2.5. Enantioselective Cyclizations

**Figure 21:**
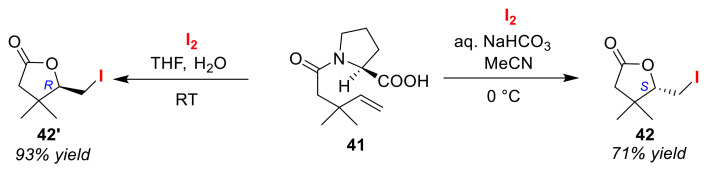
Unique enantio-controlling iodolactonization of olefinic amide **41**.

The Takano group previously reported useful synthetic intermediates for the production of trans-caronaldehydes and cis-caronaldehydes, which are important starting materials for use in the syntheses of many potent pyrethroid insecticides [66]. When (2*S*)-*N*-pent-4′-enoylproline **41** was treated with I_2_ in a mixture of MeCN and an aqueous alkaline solution, (*S*)-iodolactone **42** was obtained. However, treatment with I_2_ in aqueous THF gave the (*R*)-isomer **42′** in low enantioselectivity (Figure 21). The control mechanism of this early-reported enantioselective switch in iodolactonizations remains somewhat unclear.

**Figure 22:**
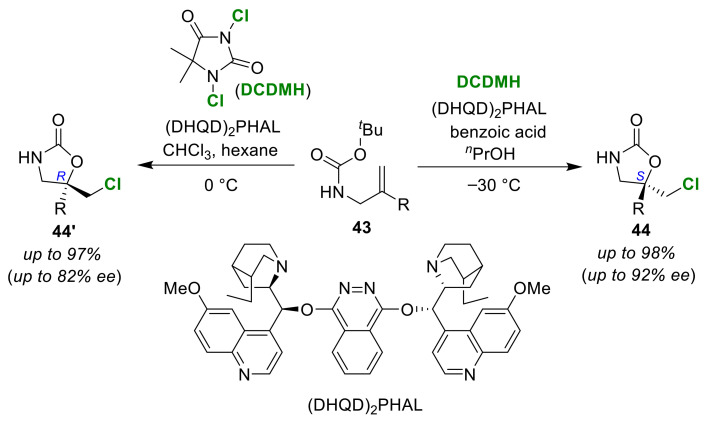
Solvent-dependent enantioselective chloro-cyclizations of olefinic carbamates **43** using (DHQD)_2_PHAL as an organocatalyst.

Borthan and co-workers developed solvent-dependent enantioselective chlorocyclizations of olefinic carbamates **43** in the presence of a hydroquinidine 1,4-phthalazinediyl diether ((DHQD)_2_PHAL) catalyst and 1,3-dichloro-5,5-dimethylhydantoin (DCDMH) [67]. When *^n^*PrOH was used as the solvent, (*S*)-form chlorolactones **44** were preferentially obtained (Figure 22). The addition of benzoic acid increased the enantioselectivity. However, this was not simply achieved by the effect of the acid. On the other hand, the use of CHCl_3_ preferentially gave (*R*)-isomers **44′**. It was confirmed that mixing hexane with CHCl_3_ resulted in good enantiomeric excesses and product yields. An enthalpy–entropy trade-off was suggested to play a central role in the prominent solvent-dependent stereo-discrimination seen in the reactions. That is, the stereoselectivity of the (*S*)-selective cyclizations in alcoholic solvent was dominated by the variation in the activation enthalpy, whereas the activation entropy governed the stereo-discrimination for the (*R*)-selective cyclizations in CHCl_3_/hexane solvent.

**Figure 23:**
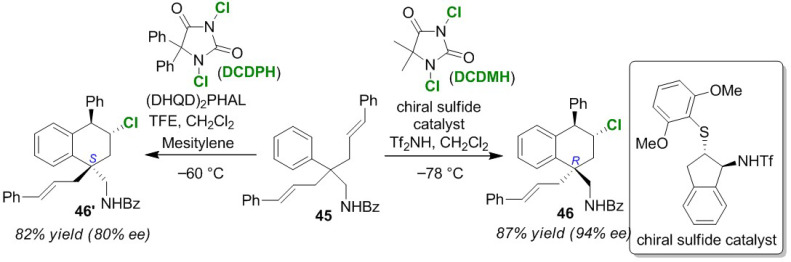
The catalyst-dependent enantioselective chlorocyclization aryl-tethered diolefin **45**.

The Zhao group developed switchable enantioselective chlorocyclizations of aryl-tethered diolefins **45** [68]. The combination of (DHQD)_2_PHAL and DCDMH was applied to the synthesis of *R* chiral hexahydrophenalene **46** (Figure 23). However, the (*S*)-isomer **46′** can be obtained by combining a chiral sulfide catalyst and 1,3-dichloro-5,5-diphenylhydantoin (DCDPH). Although the details of the reaction mechanism are still unknown, the formation of an anion bridge in the chloronium ion intermediate using a chiral sulfide catalyst is considered to enhance the stereo-selectivity.

**Figure 24:**
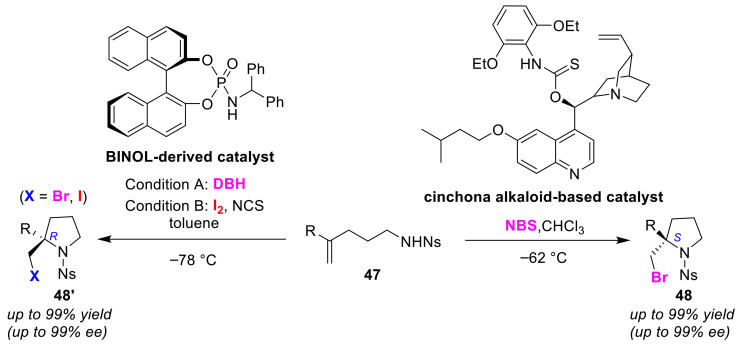
Enantioselective halo-cyclo-amination reaction of olefinic amines **47** using a cinchona alkaloid-based catalyst and BINOL-derived catalyst.

The Yeung group developed cinchona alkaloid-based chiral amino-thiocarbamate-catalyzed enantioselective bromo-amino-cyclizations [69]. In this method, (*R*)-bromocyclic amines **48** can be preferentially obtained from olefinic amines **47** (Figure 24). Chiral amino-thiocarbamates have been proposed as bifunctional catalysts, which activate both nucleophilic and electrophilic moieties in the substrate via hydrogen bonding or ion pairing. The Ishihara group reported a 1,1′-bi-2-naphthol (BINOL)-derived chiral amido-phosphate catalyst that produces (*S*)-bromo- and iodocyclic amines **48′** from olefinic amines **47** with high selectivity [70]. The key factor of the stereoselectivity in this catalyst is considered to be the asymmetrical open space with respect to the intramolecular nucleophilic moiety in the bromonium ion intermediate bound to the halogenation catalyst. In the iodo-amino-cyclization, it has been proposed that the double activation of the halogen molecule by highly active iodination species were generated from *N*-halosuccinimide and the catalyst.

### 2.6. Other Miscellaneous Reactions

**Figure 25:**
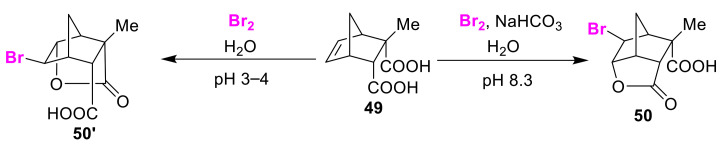
pH-dependent regioselective bromo-lactonization of olefinic dicarboxylic acid **49**.

Ranganathan and co-workers observed pH-dependent regioselectivity in the bromo-lactonization of 5-norbornene-2,3-dicarboxylic acid **49** using bromine [71]. The tertiary carboxyl bromo-lactone **50** and the secondary carboxyl isomer **50′** were synthesized by the bromo-lactonization reaction at pH 8.3 and pH 3–4, respectively (Figure 25). The pH is maintained at 3–4 by the buffering capacity of the substrate itself. The authors explained that steric bulk effects are prominently observed under acidic conditions, while the electronic p*Ka* is the controlling factor under basic conditions.

**Figure 26:**
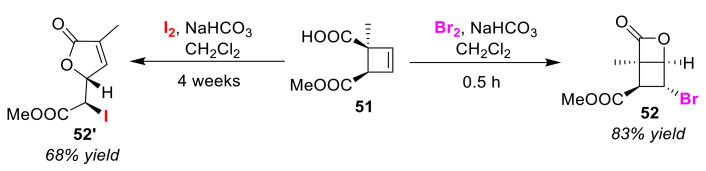
Halogen species-dependent selective halo-lactonizations of cyclo-betenoic acid **51**.

Askani and Keller developed a halogen species-dependent selective halo-lactonization of cyclobetenoic acid **51** [72]. Highly-strained bicyclo[2.2.0]lactone **52** was obtained in excellent yield by treating cyclo-betenoic acid **51** with bromine (Figure 26). Meanwhile, a five-membered lactone with an expanded ring size (**52′**) was obtained via the formation of a open chain diene upon the treatment of cyclobetenoic acid **51** with iodine. Here, the iodo-lactonization was a slow process compared to the bromo-lactonization.

**Figure 27:**
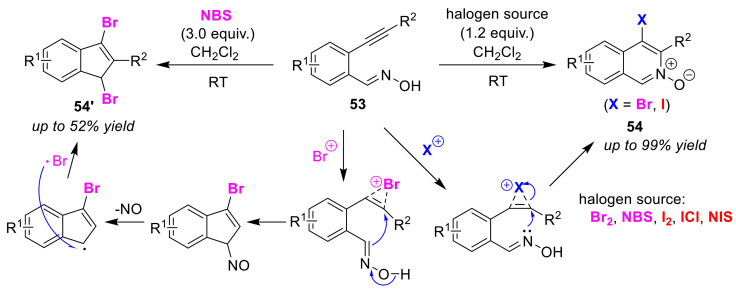
Selective synthesis of 4-haloisoquinoline *N*-oxides **54** and 1,3-dibromo-2-aryl-1*H*-indenes **54′** via halocyclizations of 2-alkynylbenzaldoximes **53**.

In 2008, Wu and Ding reported a synthetic method for 4-haloisoquinoline *N*-oxides **54** that proceeds via the endo-halocyclization of 2-alkynylbenzaldoximes **53** [73]. In terms of the electrophile used in the reaction, either using Br_2_ or NBS as bromine sources, or I_2_, ICl or NIS as iodine sources, halogenated products have been produced in good yields from many substrates (Figure 27). In 2016, the Ray group re-investigated this study. As a result, it was revealed that the use of an excess of NBS leads to the formation of unusual products, 1,3-dibromo-2-aryl-1*H*-indenes **54′**, in moderate to low yield [74]. The use of *N*-chlorosuccinimide (NCS) and NIS did not lead to these unusual products. Ray proposed a key pathway via the bromonium ion intermediate for nucleophilic attack by the *C*-center of the oxime moiety (Figure 27). However, a causal relationship between an excess of NBS and a C-center nucleophilic attack has not yet been explained.

**Figure 28:**
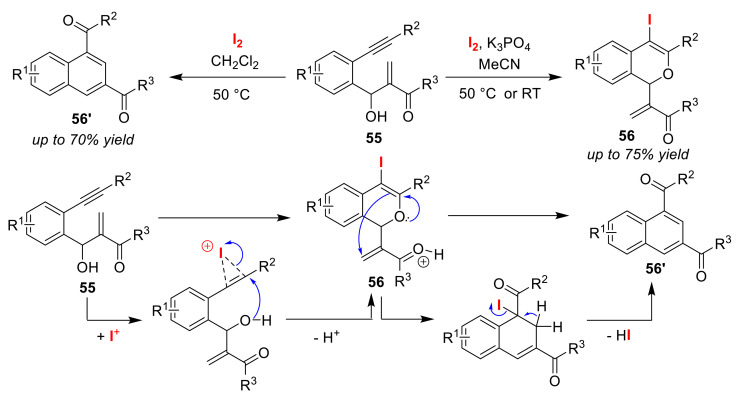
Selective synthesis of iso-chromene derivatives **56** and iodo-substituted naphthyl ketone derivatives **56′** via the halo-cyclization of 2-(2-phenylethynyl) Morita–Baylis–Hillman adducts **55** using I_2_.

The Yao group reported a method for the selective synthesis of iodo-substituted isochromene derivatives **56** and naphthyl ketone derivatives **56′** via the iodo-cyclization of 2-(2-phenylethynyl) Morita-Baylis-Hillman adducts **55** using I_2_ [75]. Iodo-substituted isochromene derivatives **56** were obtained using I_2_ and K_3_PO_4_, whereas naphthyl ketone derivatives **56′** were formed upon heating in the presence of I_2_ (Figure 28). For the synthesis of naphthyl ketone derivatives **56′**, they proposed a mechanistic pathway involving iodo-substituted isochromene derivatives **56** as the intermediates. The reason for the suppression of the conversion of isochromenes **56** to naphthalenes **56′** may be that the protons catalyzing the Michael addition of α,β-unsaturated ketone moieties are removed by the addition of bases.

## 3. Substrate-Switchable Cyclizations

As a chemical reaction progresses, the structure of the substrate dictates its fate and reactivity. In order to explore a new avenue, the substrate structure should be appropriately modified in the preceding step before any further reactions are carried out. For example, partial modification of substrate structures such as isomers and the introduction of protecting groups sometimes reflects the differences between the product structure and the course of the reaction. This section, thus, introduces substrate-switchable reactions that can be used to selectively synthesize two or more products via substrate control. The reaction conditions in the synthesis of each structural isomer are usually the same, except in some cases. In the case of switchable intramolecular cyclizations, the halogenating agent functions as a trigger for the generation of the reaction intermediates in which the organocatalyst used contributes toward the precise intermediate formation.

### 3.1. Endo/exo Selective Cyclizations

**Figure 29:**
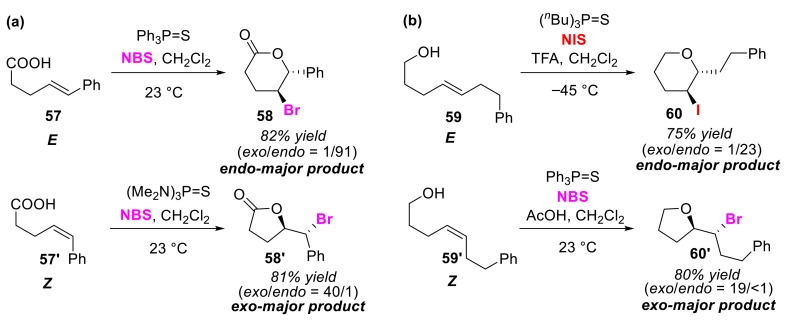
Endo/exo selective (**a**) bromo-lactonization reactions of a *E*-olefinic and *Z*-olefinic acids (**57** and **57′**) and (**b**) halo-cyclo-etherification reactions of a *E*-alkenols and *Z*-alkenols (**59** and **59′**).

Denmark and Burk showed that Lewis base catalysts containing sulfur and phosphorus atoms dramatically accelerate the reaction rate of halocyclizations of olefinic acids and alkenols using NBS and NIS as halogen sources [76]. In addition, they realized high endo/exo selectivity in bromolactonizations and halocycloetherificaions depending on the cis/trans regio-differences of the substrates. In a substrate that has a conjugated substituent on the alkene moiety, endo-cyclization is preferred over exo-cyclization due to the high stability of the positive charge localized on the benzylic carbon. However, exo-cyclized product **58′** was preferentially obtained by applying (Me_2_N)_3_PS in the bromolactonization of *Z*-olefinic acid **57′** (Figure 29a). (Me_2_N)_3_PS was used to reduce the positive charge localized on the electrophilic carbon. However, in the bromo-cyclo-etherification of a substrate without a conjugated substituent, exo-cyclization of *Z*-alkenols **59′** was preferential over endo-cyclization. To this end, the application of (*^n^*Bu)_3_PS and I_2_ to the iodocycloetherification of *E*-alkenols **59** was valid in the selectivity of endo-cyclization (Figure 29b).

**Figure 30:**
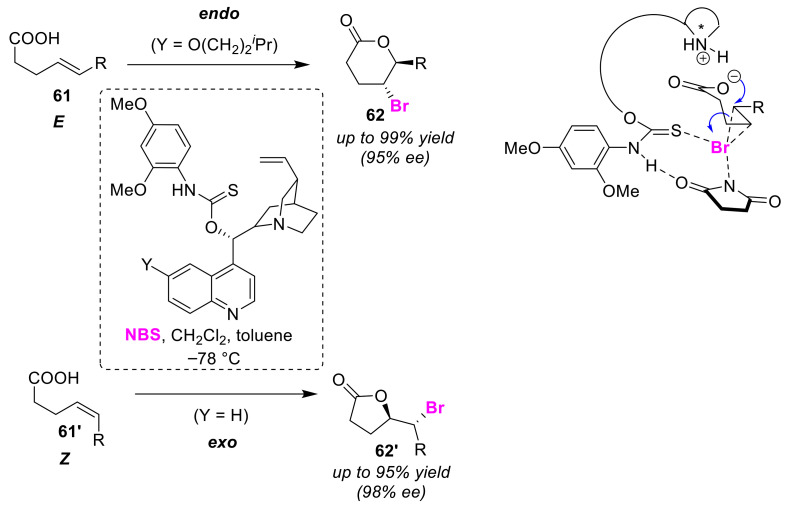
Endo/exo selective bromo-lactonizations of monosubstituted *E*-olefinic and *Z*-olefinic acids (**61** and **61′**) using an amino-thiocarbamate catalyst.

Yeung et al. developed a highly effective amino-thiocarbamate organocatalyst for the selective bromo-lactonization of monosubstituted olefinic acids. This catalyst showed high endo/exo selectivity and enantioselectivity depending on the regiostructure of the substrate. In bromo-lactonization using an amino-thiocarbamate catalyst and NBS, *E*-form olefinic acids **61** were converted to endo-cyclized products **62** [77], while *Z*-isomers **61′** produced exo-cyclized products **62′** [78]. The authors speculated that this catalyst is essentially bifunctional. The thiocarbamate moiety thus activates NBS and the quinidine moiety enhances the nucleophilicity of the carboxyl group in the substrate (Figure 30).

**Figure 31:**
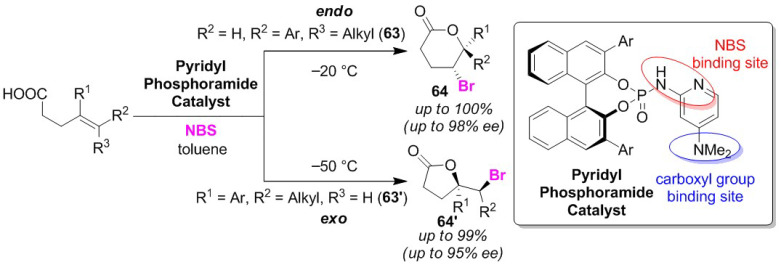
Endo/exo selective bromo-lactonizations of disubstituted olefinic acids (**63** and **63′**) using a pyridyl phosphoramide catalyst.

Meanwhile, Hara and co-workers developed a spyridyl phosphoramide catalyst that is effective for the selective bromo-lactonization of disubstituted olefinic acid. This organocatalyst furnished high endo/exo-selectivities and enantio-selectivities depending on the substituents present in the substrate [79]. Whether endo-selectivities or exo-selectivities are promoted in reactions potentially relies on the position of the aryl group as the conjugated substituent attached to the alkene moiety to enhance the stability of the localized positive charge. In the same study, 6-arylsubstituted and 5-arylsubstituted olefinic acids (**63** and **63′**) were selectively obtained as endo-cyclized and exo-cyclized products (**64** and **64′**), respectively (Figure 31). This result implies that the endo/exo selectivity corresponds to the bonding position of the aryl group. In plausible reaction mechanisms, the dimethylamine moiety and another nitrogen atom in the pyridyl phosphoramide catalyst were proposed as a carboxyl group and NBS binding sites. Furthermore, the results show that the brominating reagent affects the enantioselectivity of the reaction, which suggested that the brominating agent plays a role in the transition state.

**Figure 32:**
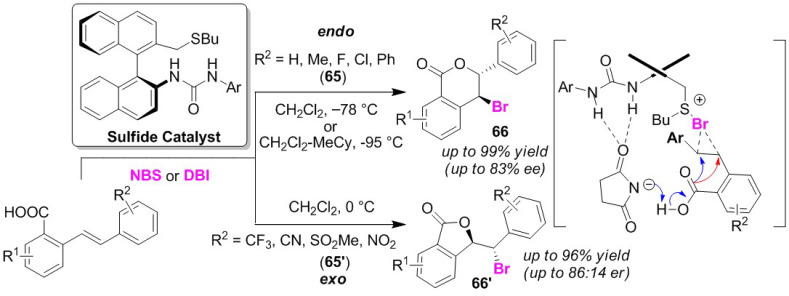
Endo/exo selective bromo-lactonizations of stilbene-type carboxylic acids (**65** and **65′**) using a chiral sulfide catalyst.

Shirakawa and co-workers developed an efficient chiral sulfide organocatalyst for the selective bromo-lactonization of a stilbene-type carboxylic acid. This catalyst showed high endo/exo selectivity and moderate enantioselectivity depending on the substituents present in the substrates. Substrates possessing electron-donating substituents at the non-nucleophilic aryl moiety (**65**) undergo endo-cyclization [80], while those possessing electron-withdrawing substituents (**65′**) undergo exo-cyclization [81]. This endo/exo selectivity was, thus, determined to be governed by the electronic factors of the aryl moiety. The sulfide catalyst is bifunctional, with a urea site for binding to the brominating reagents such as NBS and dibromoisocyanuric acid (DBI), as well as a sulfide site that can promote bromination (Figure 32). The authors proposed that bromosulfonium species and a Brønsted base are simultaneously generated via the rearrangement of the bromonium ion to the sulfide site in a complex of the brominating agent with the catalyst, effectively resulting in an intramolecular nucleophilic reaction. The chiral sulfide catalyst also contributes to the high degree of control of the enantio-selectivity.

**Figure 33:**
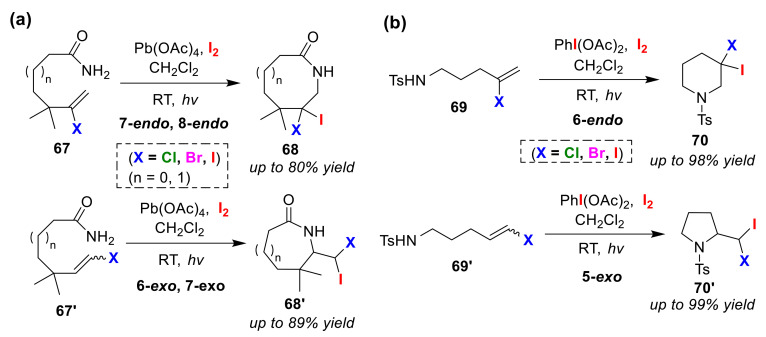
Endo/exo selective (**a**) halolactamization reactions of halovinyl amides (**67** and **67′**) and (**b**) halo-amino-cyclization reactions of halovinyl sulfonamides (**69** and **69′**) via intramolecular halogen control.

Li and co-workers developed an amidyl radical cyclization reaction for the regioselective halolactamizations of halovinyl amides (**67** and **67′**) and the regioselective haloaminocyclizations of halovinyl sulfonamides (**69** and **69′**). Usually, generation of an amidyl radical results in a decrease in regioselectivity. However, the radical-stabilizing effect of a vinylic halogen substitute allows high endo/exo regioselectivity to be realized. In the halolactamization [82] and haloaminocyclization reactions [83], the authors used Pb(OAc)_4_ and PhI(OAc)_2_, respectively. These reactions are started with photo-stimulation in the presence of I_2_ (Figure 33). Removal of the halogen substituent from the substrate resulted in reduced regioselectivity and contamination of the lactone product formed via the ionic mechanism.

**Figure 34:**
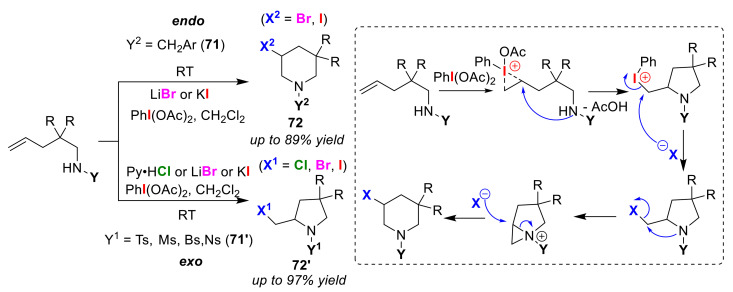
Endo/exo selective halo-amino-cyclization reactions of olefinic amines **71** and sulfonamides **71′** using a hypervalent iodine reagent.

Li and Liu developed the regioselective halo-amino-cyclizations of olefinic amines (**71** and **71′**) using a hypervalent iodine reagent, PhI(OAc)_2_. In this method, endo/exo regioselectivity was controlled by the amine protection groups [84]. When a benzyl group or its derivative was used as a protecting group, endo-cyclized products **72**, were selectively produced, while the use of a sulfonamide-based protecting group selectively gave exo-cyclized products **72′** (Figure 34). PhI(OAc)_2_ plays a role in activating the olefinic moiety. It was determined that, in this method, any halogen atom can be easily introduced using an arbitrary inorganic halogen salt. In the endo-cyclization process, the authors showed that the reaction mechanism proceeds via an exo-cyclization intermediate. The rate-determining factor in the endo-cyclized ring was, thus, interpreted to be related to the formation of a bicycle intermediate during dehalo-cyclizations.

**Figure 35:**
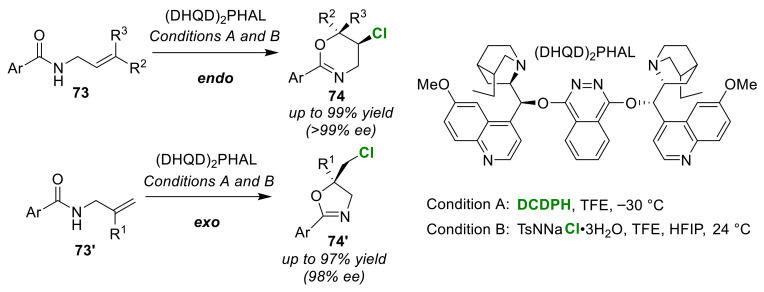
Selective syntheses of oxazines and oxazolines via the chloro-cyclization of olefinic amides (**73** and **73′**) using (DHQD)_2_PHAL.

Borhan et al. used (DHQD)_2_PHAL in the chloro-cyclizations of olefinic amides (**73** and **73′**), and reported a selective synthetic method for oxazines **74** and oxazolines **74′**. The use of DCDPH as a halogen source in trifluoroethanol solvent required low temperature conditions [85], and the use of TsNNaCl trihydrate enabled the reactions to be performed at room temperature [86]. Both methods afforded the products in excellent yields with high enantioselectivities. The endo-cyclized products **74** and exo-cyclized products **74′** were selectively obtained from disubstituted butanoic amides **73** and monosubstituted butanoic amides **73′**, respectively (Figure 35).

**Figure 36:**
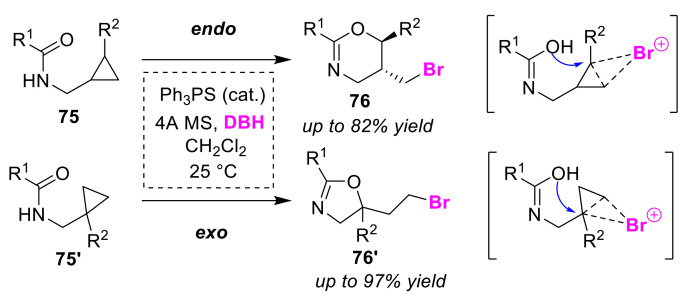
Heterocycle formations of oxazines **76** and oxazolines **76′** from cyclopropyl amides (**75** and **75′**) via bromocyclization reactions using a Lewis basic sulfide catalyst.

The Yeung group developed the selective synthesis of oxazines **76** and oxazolines **76′** using a Lewis base sulfide catalyst for the bromocyclizations of cyclopropyl amides (**75** and **75′**). In this method, 1,3-dibromo-5,5-dimethylhydantoin (DBH) can be used as a halogen source at room temperature, and the endo/exo selectivity is dependent on the regio-isomeric structure of the substrate [87]. Using 1,2-substituted cyclopropyl amides **75** and 1,1-substituted cyclopropyl amides **75′**, the oxazines **76** via endo-cyclized products and oxazolines **76′** via exo-cyclized products were selectively obtained, respectively (Figure 36). The cyclizations were not inhibited, even for reactions carried out in the presence water. However, the reactions were carried out under dehydrated conditions in the presence of 4Å molecular sieves. The authors explained the reaction mechanism, in which bromonium-like or carbocation intermediates generated by activation of a Lewis base sulfide catalyst–bromo cation complex at the cyclopropane moiety are nucleophilically attacked by an amide moiety.

**Figure 37:**
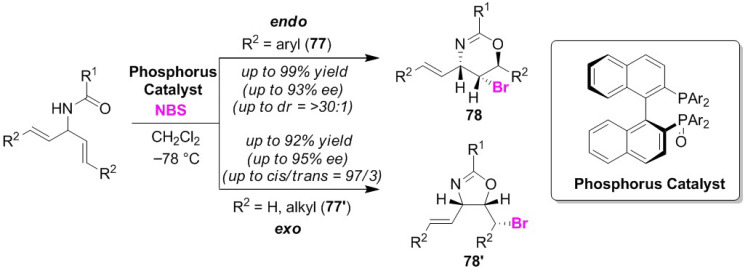
Selective synthesis of oxazines **78** and oxazolines **78′** via the bromocyclizations of dienyl amides (**77** and **77′**) using DTBM-BINAP monoxide.

Hamashima and Kawato developed a useful phosphorous catalyst for the selective bromocyclizations of dienyl amides (**77** and **77′**). The use of di-*tert*-butylmethoxyphenyl (DTBM) -2,2′-bis(diphenylphosphino)-1,1′-binaphthyl (BINAP) monoxide as a phosphorous catalyst in the presence of NBS provided the desired cyclization products in excellent yields with high enantioselectivities [88]. The controlling factor of this endo/exo selectivity was determined to be the substituent of the olefinic moiety in the substrate, and, thus, aryl substituents and alkyl phosphates gave oxazines **78** as endo-cyclized products and oxazolines **78′** as exo-cyclized products, respectively (Figure 37). The authors proposed a reaction mechanism in which the Lewis basic site, P=O, activates NBS and the resulting P^+^OBr moiety contributes to the chiral transfer of a bromo cation to the olefinic moiety of the substrate. This involves the independent activation of the olefinic moiety by the phosphorus catalyst. Their study is, to the best of our knowledge, the first successful de-symmetrization of prochiral dienyl amides.

### 3.2. O/N Atom-Selective Cyclizations

**Figure 38:**
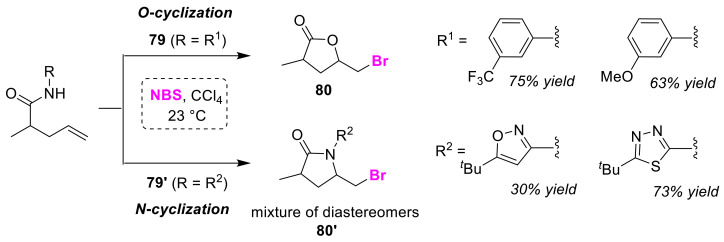
Classical O/N atom-selective bromocyclizations of olefinic amides (**79** and **79′**).

In 1989, Brinkmeyer and co-workers tried to develop a method for constructing the lactam ring based on bromo-lactonization using NBS. The lactams **80′** were obtained from bromo-lactonization of olefinic amides bearing heterocyclic aromatics as nitrogen substituents (**79′**). However, changing the nitrogen substituents to carbocyclic aromatics from heterocyclic aromatics gave unexpected lactones **80** (Figure 38) [89]. The authors concluded these results to be failures in terms of lactam formation because the lactones are *O*-cyclized products that have undergone hydrolysis. This study represents a pioneering example of O/N atom-selective cyclizations.

**Figure 39:**
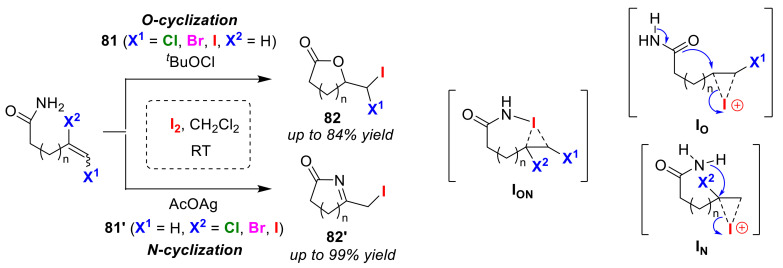
Intramolecular halogen substituent-dependent O/N atom-selective iodo-amino-cyclizations of the olefinic amides **81** and **81′**.

The Li group reported a convenient and efficient method for O/N atom-selective iodo-cyclizations of olefinic amides under non-basic conditions. Internal vinylic halogen substituent directed *O*-cyclization of olefinic amides **81** and terminal vinylic halogen substituent directed *N*-cyclization of substrates **81′** gave iodolactones **82** and iodoiminolactam **82′**, respectively [90]. The fact that the *O*/*N* selectivity can be controlled according to the position of the halogen atom substituent is particularly noteworthy (Figure 39). The authors concluded that the halocyclization of olefinic amides is likely to proceed via iodonium ion transfer. As a plausible mechanism, the authors proposed that there is the oxidative formation of an N–I bond followed by hetero-cleavage and transfer of an intramolecular iodonium ion to a double bond to generate a bridged iodonium intermediate, **I_ON_**. An important factor that contributes to the *O*/*N* selectivity is the effect of lone pair repulsion. When the nucleophilic moiety in the molecule approaches the internal carbon atom of the halogen-bonded iodonium intermediate **I_N_**, it experiences lone pair repulsion with the halogen atom. *N*-cyclization was preferential in the intramolecular cyclization reaction, involving an internal vinylic halogen substituent. This is because the repulsion of the lone pair with the oxygen atom is much stronger than that with the nitrogen atom. However, *O*-cyclization is usually preferential in the intramolecular nucleophilic reaction of the terminal halogen-bonded iodonium intermediate, **I_O_**, because the interaction between the halogen substituent and the nitrogen or oxygen at the nucleophilic site can be ignored.

**Figure 40:**
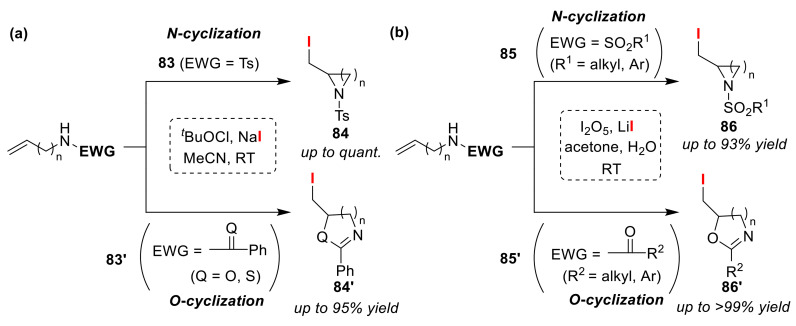
Selective syntheses of cyclic amines with or without the incorporation of other heteroatoms (**84**, **84′**, **86**, and **86′**) via the iodo-cyclizations of olefinic *N*-protected amines (**83**, **83′**, **85**, and **85′**).

Minakata and Komatsu et al. achieved the selective synthesis of cyclic amines and azolines via iodo-cyclizations of *N*-substituted olefinic amines using *^t^*BuOI in situ generated from the reaction between *^t^*BuOCl and NaI (Figure 40a) [91,92]. *N*-tosyl substituted olefinic amines **83** gave cyclic amines **84** of various ring sizes, while *N*-acyl and *N*-thioacyl substituted olefinic amines **83′** instead produced azolines (**84′**) by incorporating two heteroatoms in the structures, allowing the formation of oxazolines, oxazines, thioxazolines, and thioxazines. The authors proposed that the iodonium ion of the *N*-sulfonamide intermediate transfers to the olefin moiety in the substrates. Similar heterocycles can be obtained using a chloramine-T/I_2_ system. In addition, the Liu group recently reported an eco-friendly protocol based on an I_2_O_5_/LiI system (Figure 40b) [93]. The use of this method allows a reduction in the reaction time and leads to an improvement in the yield.

**Figure 41:**
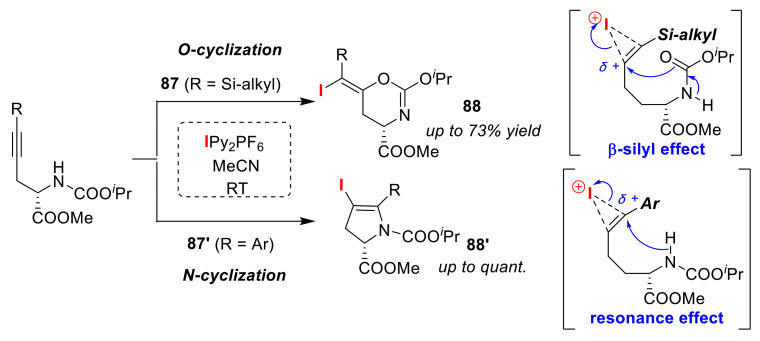
Selective syntheses of oxazines **88** and 2,3-dihydropyrroles **88′** via iodo-cyclizations of alkylamides (**87** and **87′**) using IPy_2_PF_6_.

As a selective heterocycle synthesis in the iodocyclization of alkylamides, the Wada group developed a method for *O*/*N* atom-selective cyclizations controlled by the substituent of the alkyne moiety. Treatment of silyl- (**87**) and aryl-functionalized substrates (**87′**) with IPy_2_PF_6_ at room temperature led to the formation of oxazines **88** [94] and 2,3-dihydropyrroles **88′** [62], respectively. The authors explained that the controlling factor of the O/N selectivity in these iodo-cyclizations was due to the both β-silyl and resonance effects (Figure 41).

### 3.3. Ene/Diene Selective Cyclizations

**Figure 42:**
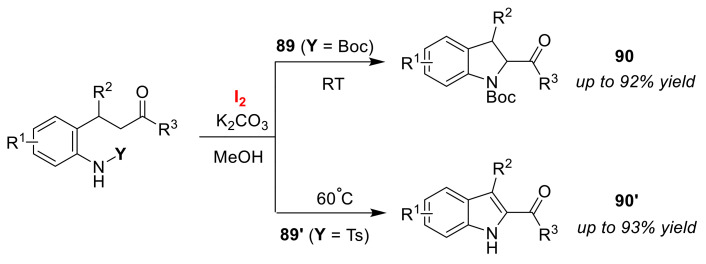
Selective syntheses of 2-acylindolines **90** and 2-acylindoles **90′** via the *N*-protection- dependent cyclization of *O*-acylethyl *N*-subsituted anilines (**89** and **89′**) using an I_2_/K_2_CO_3_ system.

The Zhang group developed the selective synthesis of 2-acylindolines **90** and 2-acylindoles **90′** via the cyclizations of *o*-acylethyl *N*-substituted anilines **89** and **89′** using an I_2_/K_2_CO_3_ system [95]. The selectivity of both routes was dependent on the *N*-protection groups, and, as a result, *N*-Ts and *N*-Boc protections furnished 2-acylindolines **90** and 2-acylindoles **90′**, respectively (Figure 42). The authors proposed a reaction mechanism involving the α-iodoketone generated via the intramolecular N–I intermediate, which then contributes to the intramolecular nucleophilic substitution reaction. The *N*-tosyl cyclized intermediate was further deprotected and, finally, converted to 2-acylindoles **89′**.

**Figure 43:**
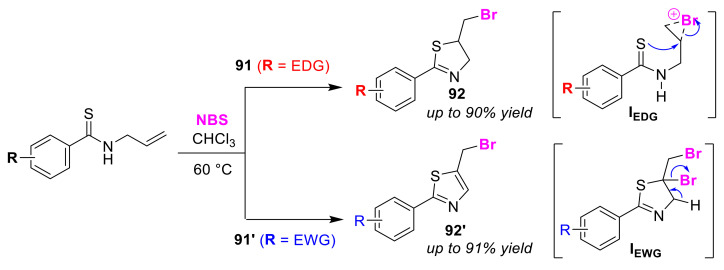
Selective syntheses of thiazolines **92** and thiazoles **92′** via bromocyclizations of functionalized *N*-allylbenzenethioamides (**91** and **91′**) using NBS.

The Pan group described the selective synthesis of thiazolines **92** and thiazoles **92′** via intramolecular cyclizations of *N*-allylbenzothioamides **91** and **91′** using NBS [96]. The electron-donating group (EDG) and electron-withdrawing group (EWG) attached to the aromatic moieties in the substrates led to different products, thiazolines **92** and thiazoles **92′**, respectively (Figure 43). The thiazoline formation was explained on the basis of an intramolecular nucleophilic substitution occurring that involves the strongly nucleophilic sulfur atom in the bromonium ion intermediate, **I_EDG_**. However, it was interpreted that the formation of thiazoles **92′** was, as a result of the elimination of the dibromothiazoline intermediate, **I_EWG_**. In the reaction mechanism, the intermediate is generated by the overreaction of thiazolines **92**, and the authors proposed that the hydrogen in the C–H bond adjacent to the sulfur atom can be easily bromo-substituted via an EWG effect.

### 3.4. Syn/Anti (Cis/Trans) Selective Cyclizations

**Figure 44:**
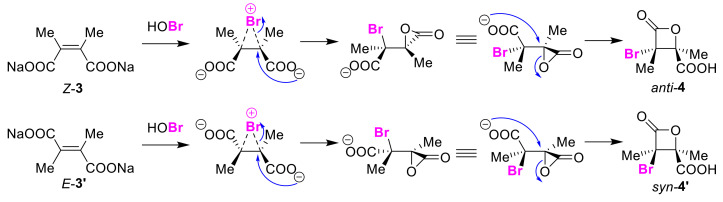
Early-reported stereoselective halo-lactonization of the sodium salts of dimethyl maleic acid *Z*-**3** and dimethyl fumaric acid *E*-**3′**.

In 1937, Tarbell and Bartlett reported the first example of β-lactone formation via a halo-lactonization reaction [22]. The chlorolactone obtained from the chlorination of dimethylmaleic acid sodium salt *Z*-**3** was different in the melting point from the chlorolactone produced by a similar treatment of dimethylfumarate sodium salt *E*-**3′**. Thus, different bromo-lactones were produced using a similar preparation involving bromine. This unique preparation was interpreted as an intramolecular S_N_1-type nucleophilic reaction with the adjacent carboxy group that proceeded after carbocation generation. The obtained product was described as a stereoisomer, but its stereostructure was not made clear. If the halonium ion is considered as the intermediate in the halo-lactonization reaction, the sodium salts of *Z*-**3** and *E*-**3′** give syn-**4** and anti-lactone **4′**, respectively. Apart from the previously mentioned report, in 2001, the William group reinvestigated this work and showed the opposite result to the previous interpretation [23]. That is, the X-ray crystal structure analysis of the stereoisomers revealed that the use of dimethyl maleic acid *Z*-**3** and dimethyl fumaric acid *E*-**3′** sodium salts resulted in anti-**4** and syn-lactone **4′** being produced. Based on this new result, the authors suggested a new reaction mechanism that proceeds via a three-membered cyclic lactone intermediate (Figure 44).

**Figure 45:**
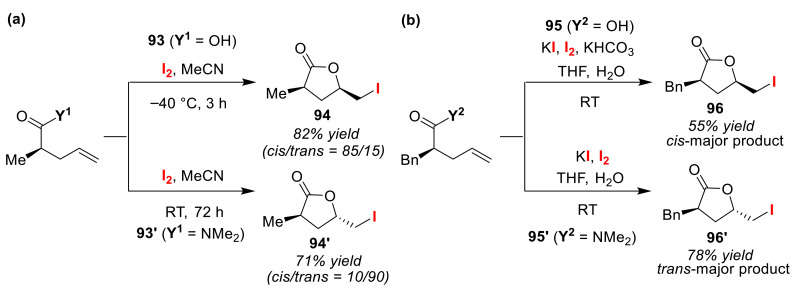
Cis/trans stereoselective iodo-lactonizations of olefinic acids (**93** and **95**) and amides (**93′** and **95′**) using I_2_.

Rao et al. reported the cis/trans-selective synthesis of iodolactone, which is a key intermediate in the synthetic study of the C_12_ polyketide unit (C1–C8) of jaspamide and geodiamolides A–F [97]. While cis-iodolactone **94** was obtained as the main product from the treatment of olefinic acid **93** with I_2_ at −40 °C, olefinic *N*,*N*-dimethylamide **93′** preferentially reacted at room temperature to give the trans-isomer **94′** (Figure 45a). The reaction times required for both cases were also very different. For the synthesis of useful HIV-1 protease inhibitors, Trova et al. attempted the selective asymmetric synthesis of cis-lactones and trans-lactones via iodolactonization [98]. Their method relied on the reactivity differences of the substrates used to determine the selectivities of the reactions. Thus, olefinic acid **95** was treated with KI/I_2_/KHCO_3_ to give cis-lactone **96** as the main product, while treatment of olefinic *N*,*N*-dimethylamide **95′** with KI/I_2_ selectively led to the formation of trans-lactone **96′** (Figure 45b). Unfortunately, the control mechanism of the cis/trans selectivity in these reactions remains unclear.

**Figure 46:**
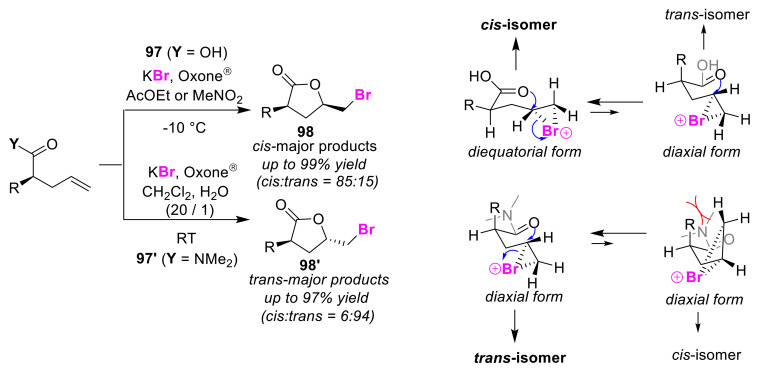
Stereoselective bromo-lactonizations of olefinic acids **97** and amides **97′** using a KBr/Oxone^®^ system.

Togo et al. developed the selective asymmetric synthesis of cis-lactones and trans-lactones (**98** and **98′**) via oxidative bromo-lactonization [99]. This method also exploited the functional differences between the substrates to determine the selectivities of the reactions. Under KBr/Oxone^®^ conditions, olefinic acid **97** gave cis-lactone **98** as the main product, while olefinic *N*,*N*-dimethylamide **97′** preferentially produced trans-lactone **98′** (Figure 46). It was shown that the effect of the solvent on the cis/trans selectivity is negligible [100]. In order to explain the selectivity of each of the reactions, Togo et al. suggested the following reaction mechanism based on the calculation of several transition states involved in the iodo-lactonization processes by Kürth research [101]. In the cis-selective reaction of olefinic acids **97**, a highly stable diequatorial form of the reactant was preferred over the diaxial form, whereas, in the trans-selective reaction of olefinic amides **97′**, the diaxial form in which the substituent adjacent to the dimethylamine moiety does not hinder the electrophilic reaction site was preferred. This selective asymmetric synthesis of cis-lactone **98** was used to achieve the first total synthesis of (+)-dubiusamine C.

**Figure 47:**
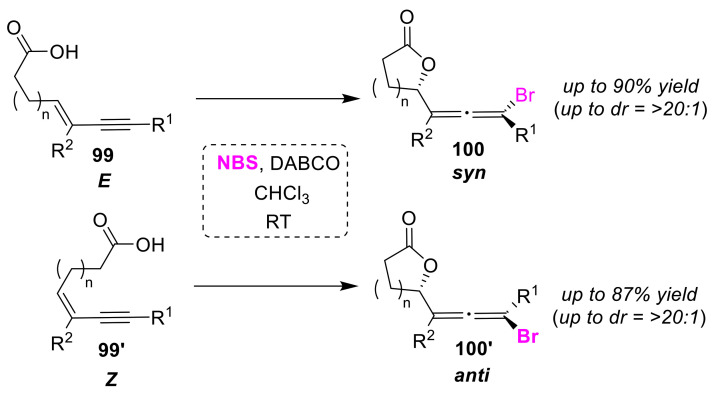
Stereoselective bromo-lactonizations of *E*- and *Z*-enynyl acids (**99** and **99′**) using DABCO.

The Tang group developed the bromolactonizations of enynyl acids using 1,4-diazabicyclo[2.2.2]octane (DABCO) as a Lewis base catalyst [102]. This method, useful for the synthesis of multi-substituted chiral allenyl lactones, can be used to convert *E*-enynyl and *Z*-enynyl acids (**99** and **99′**) to syn-bromoallenyl and anti-bromoallenyl lactones (**100** and **100′**), respectively (Figure 47). Perfect enantioselectivity (>99% ee) was achieved for the substrates with chiral substituents. Furthermore, the authors designed a cinchona alkaloid derivative with a tosyl urea group for use as an organocatalyst, which, when used in an enantioselective bromolactonization reaction on a prochiral substrate, successfully produces syn-bromoallenyl lactone from *Z*-enine [103].

**Figure 48:**
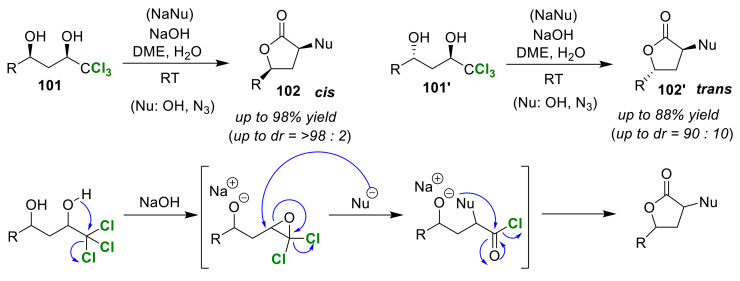
Stereoselective dechlorolactonization reaction of syn-trichlorodiols and anti-trichlorodiols (**101** and **101′**) under basic conditions.

Snowden et al. developed the cis/trans selective dechloro-lactonization of 1-trichloromethyl-1,3-diol, which proceeded under basic conditions [104]. Their method stereoselectively produced cis- disubstituted and trans-disubstituted lactones (**102** and **102′**), respectively, from syn- and anti-1,3-diols (**101** and **101′**), in excellent yields (Figure 48). In this reaction, the intramolecular cyclization and intermolecular nucleophilic addition reactions were continuous, and either a hydroxyl group or an azide can be introduced as a nucleophile. A conceptual reaction mechanism was proposed in which an epoxide intermediate generated by dechlorination undergoes epoxide ring opening via external nucleophilic attack to generate an acyl chloride, which is an active species involved in lactonization.

### 3.5. Enantioselective Cyclizations

**Figure 49:**
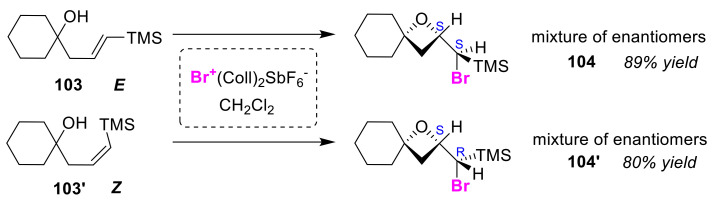
Stereoselective bromocycloetherification of *E*-silyl and *Z*-silyl homoallylic alcohols (**103** and **103′**) using Br^+^(Coll)_2_SbF_6_^−^.

Rousseau et al. reported the exo-bromocycloetherification of *E*-silylhomoallylic alcohol **103** and its *Z*-isomer **103′** using bis(sym-collidine)bromine(I) hexafluoroantimonate (Br^+^(Coll)_2_SbF_6_^-^) [105]. In the related 4-exo cyclization reaction, there was the problem of the competing 5-endo cyclization, while the introduction of a silyl group to the olefinic moiety contributed toward the high selectivity of the reaction. The cyclization of unsubstituted silyl allylic tertiary alcohols with Br^+^(Coll)_2_SbF_6_^-^ gave the corresponding single oxetane diastereo-isomers. As a result, (*S*,*S*)- and (*R*,*R*)-oxetane enantiomers **104**, as well as (*S*,*R*)- and (*R*,*S*)-oxetane enantiomers **104′**, were obtained from *E*-silyl homoallylic alcohol **103** and its *Z*-isomer **103′**, respectively (Figure 49). It is possible to utilize this method for the formation of iodooxetane, but it has been found to result in low product yields.

**Figure 50:**
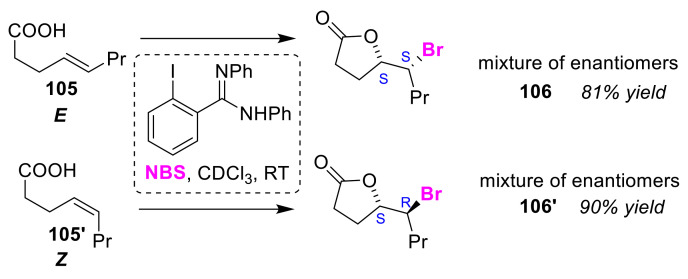
Stereoselective bromo-lactonizations of *E*-olefinic and *Z*-olefinic acids (**105** and **105′**) using *o*-amidinyl-substituted iodobenzene as an organocatalyst.

In 2006, Braddock et al. developed the first organocatalytic method to transfer electrophilic bromine to olefins [106]. The electrophilic bromo-iodane intermediate generated by the reaction of *o*-amidinyl-substituted iodobenzene is believed to function as an organocatalyst using NBS as a bromine source, activating the substrate olefinic moiety. This pioneering study also detailed the bromo-lactonization of *E*-olefinic acid **105** and its *Z*-isomer **105′** to give the enantiomers (*S*,*S*)-lactones and (*R*,*R*)-lactones **106**, and (*S*,*R*)- and (*R*,*S*)-lactones **106′**, respectively (Figure 50).

**Figure 51:**
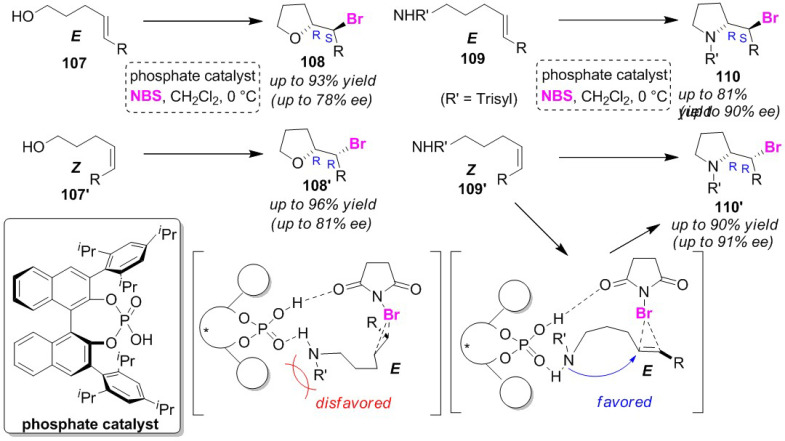
Enantioselective bromocyclizations of *E*-olefinic and *Z*-olefinic alcohols (**107** and **107′**) and amines (**109** and **109′**) using a phosphate catalyst.

A chiral phosphate organocatalyst developed by Shi group was used to conduct enantioselective *O*-bromocyclizations and *N*-bromocyclizations [107]. Bromocyclization of *E*-olefinic and *Z*-olefinic alcohols (**107** and **107′**) and amines (**109** and **109′**) enantio-selectively produced *exo*-cyclized tetra-hydrofurans (**108** and **108′**) and pyrrolidines (**110** and **110′**), respectively (Figure 51). The disadvantage of the reactions is that they require long reaction times of around three days. The authors proposed that the phosphate site of this catalyst can activate both the electrophile (NBS) and nucleophiles (substrates) via hydrogen bonding. In the bromocyclization of olefinic amines, hydrogen bonding and the steric interaction of the *N*-protecting group can be interpreted as contributing to the orientation of the substrates. Denmark and Burk independently developed the same catalyst, and reported a method that can be used for the enantioselective bromo-cyclo-etherification of olefinic alcohols in a relatively short time in which the catalytic mechanism is different from that reported by the Shi group [108].

### 3.6. Other Miscellaneous Reactions

**Figure 52:**
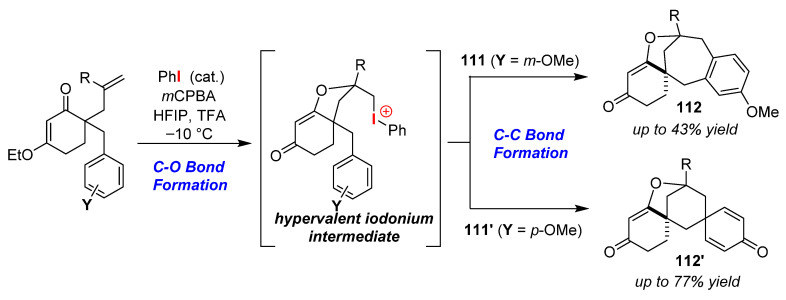
Selective synthesis of oxabicyclic [4.2.1]nonanes **112** and [3.2.1]octanes **112′** mediated by a hypervalent iodonium intermediate.

Lupton and co-workers reported a method for the selective synthesis of oxabicyclic [4.2.1]nonanes **112** and [3.2.1]octanes **112′** catalyzed by iodobenzene via cascade C–O/C–C bond formations [109]. In this method, a bicyclo moiety was formed by treating 3-alkoxy-6-allyl-6-methylaryl cyclohexen-2-ones with the catalyst PhI/*m*CPBA in a mixed solvent of hexafluoroisopropanol (HFIP)/trifluoroacetic acid (TFA). The bicyclization reactions of *m*-methoxybenzyl and *p*-methoxybenzyl isomers (**111** and **111′**) gave oxabicyclic [4.2.1]nonanes **112** and [3.2.1]octanes **112′**, respectively (Figure 52). The selectivities of the reactions depend on the C–C bond formation step of the hypervalent iodonium intermediate generated by C–O bond formation, and it is also thought that the orientation of the aryl moiety has a remarkable influence on the reactions due to a substituent effect.

**Figure 53:**
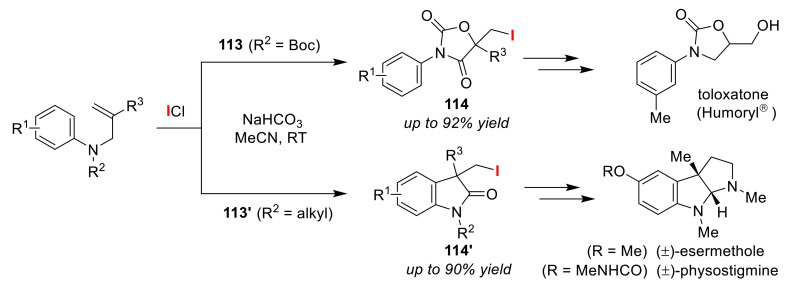
Selective synthesis of oxazolidine-2,4-diones **114** and oxindoles **114′** via the protection group dependent selective iodo-cyclizations of *N*-protected *N*-aryl-acrylamides **113** and **113′**.

The Liu group recently reported the protection group dependent selective iodo-cyclizations of *N*-protected *N*-aryl-acrylamides using ICl in the presence of NaHCO_3_ [110]. Oxazolidine-2,4-diones **114** were obtained by exo-type iodolactonizations of *N*-Boc *N*-arylacrylamides **113**, while oxindoles **114′** were prepared by exo-type iodocarbocyclizations of *N*-alkyl *N*-arylacrylamides **113′** (Figure 53). Similar treatment of *N*-Boc *N*-arylacrylamides with 1,1-disubsituted moieties led to the formation of endo-type iodo-carbo-cyclizations. In this study, the authors also demonstrated that the obtained products are useful synthetic intermediates for bioactive compounds, such as toloxatone, (±)-esermethiole, and (±)-physostigmine.

## 4. Conclusions

In this review, we have focused on controllable cyclization reactions that are very useful for the formations of skeletons from various molecules in order to further expand the versatility of halogen-dependent reactions for heterocycle synthesis. In this article, these controllable reactions are categorized into reagent-switchable and substrate-switchable reaction types. There are many benefits to these reactions that have been described herein, so it is expected that these reactions are very practical to use. All of the strategies exhibit high product selectivities and there are options in terms of reagent and substrate controls to allow flexibility in the formation of the skeletons of organic molecules.

The endo/exo selectivity of halocyclization reactions has been of interest since the middle of the 20th century with recent rapid progress in this area. Although exo-cyclization is potentially favored in halocyclization reactions, the fact that endo-cyclization can selectively proceed is a great advantage. In addition, the development of organocatalysts for use in controllable stereoselective cyclizations has become an important basis for possible further developments in this area. Regioselective and stereoselective introductions of halogen atoms are powerful tools to aid the successful construction of target molecules. The discovery of new intermediates that trigger the reactions of saturated substrates could be the key to boosting innovative syntheses. However, further efforts are needed to elucidate the reaction mechanisms mentioned in this review that remain unknown. In recent years, green chemistry-directed, recyclable hypervalent iodine reagents and catalysts have been under rapid development. As a future topic, their further contribution to halogen-controlled intramolecular cyclizations is strongly desired. Therefore, this review provides appropriate literature and concepts for synthetic chemists engaged in heterocycle synthesis and for brave pioneers in next-generation reaction development in organic synthesis.
